# Malignant T cells express lymphotoxin α and drive endothelial activation in cutaneous T cell lymphoma

**DOI:** 10.18632/oncotarget.3837

**Published:** 2015-04-15

**Authors:** Britt Lauenborg, Louise Christensen, Ulrik Ralfkiaer, Katharina L. Kopp, Lars Jønson, Sally Dabelsteen, Charlotte M. Bonefeld, Carsten Geisler, Lise Mette R. Gjerdrum, Qian Zhang, Mariusz A. Wasik, Elisabeth Ralfkiaer, Niels Ødum, Anders Woetmann

**Affiliations:** ^1^ Department of International Health, Immunology and Microbiology, University of Copenhagen, Copenhagen, Denmark; ^2^ Department of Hematology, Copenhagen University Hospital, Copenhagen, Denmark; ^3^ Center for Genomic Medicine, Copenhagen University Hospital, Copenhagen, Denmark; ^4^ Department of Oral Medicine and Pathology, School of Dentistry, University of Copenhagen, Copenhagen, Denmark; ^5^ Department of Pathology, Roskilde Hospital, Roskilde, Denmark; ^6^ Department of Pathology and Laboratory Medicine, University of Pennsylvania, Philadelphia, PA, USA; ^7^ Department of Pathology, Copenhagen University Hospital, Copenhagen, Denmark

**Keywords:** CTCL, LTA, angiogenesis

## Abstract

Lymphotoxin α (LTα) plays a key role in the formation of lymphatic vasculature and secondary lymphoid structures. Cutaneous T cell lymphoma (CTCL) is the most common primary lymphoma of the skin and in advanced stages, malignant T cells spreads through the lymphatic to regional lymph nodes to internal organs and blood. Yet, little is known about the mechanism of the CTCL dissemination. Here, we show that CTCL cells express LTα *in situ* and that LTα expression is driven by aberrantly activated JAK3/STAT5 pathway. Importantly, via TNF receptor 2, LTα functions as an autocrine factor by stimulating expression of IL-6 in the malignant cells. LTα and IL-6, together with VEGF promote angiogenesis by inducing endothelial cell sprouting and tube formation. Thus, we propose that LTα plays a role in malignant angiogenesis and disease progression in CTCL and may serve as a therapeutic target in this disease.

## INTRODUCTION

Cutaneous T cell lymphomas (CTCLs) are a group of lymphoproliferative disorders characterized by a clonal expansion of T cells primarily in the skin. Mycosis fungoides (MF) is the most common type of CTCL and accounts for about 50% of all patients with cutaneous lymphomas [1]. Classical MF progresses through three stages - the patch stage, the plaque stage and the tumor stage. The clinical course of MF is usually indolent and the patch and plaque stages are associated with good prognosis. Accordingly, long-term follow-up studies have demonstrated that the early-stage patients have a similar life expectancy to that of control populations [2-4]. However, in some cases, the disease progresses to tumor formation and involvement of lymph nodes and internal organs. The advanced MF is often refractory to treatment and the prognosis tends to be poor [4-5]. The pathogenesis of MF remains poorly understood. However, abnormalities in cytokine production [6-10], a cytokine-independent constitutive activation of the Janus kinases (JAKs)/Signal transducer and activator of transcription (STAT) pathway [11-13] and aberrant regulation of nuclear factor κB (NFκB) [14-17] are known to play a key role in the pathogenesis of CTCL.

The *de novo* formation of blood and lymphatic vasculature was recently shown to be involved in the progression of CTCL, particularly MF [18-20]. Biopsies from patients diagnosed with CTCL demonstrated a stage-dependent increase in the mean microvessel density and malignant CTCL T cells have been shown to express several angiogenic factors, including VEGF [20]. Recently we demonstrated that the expression of VEGF by malignant CTCL cells is mediated by the aberrant activation of JAK3 and c-Jun N-terminal kinases (JNKs) [12]. Furthermore, skin biopsy tissue from patients diagnosed with Sézary syndrome, a subtype of CTCL, also revealed an increase in lymph-angiogenesis as measured by an increased staining for podoplanin (PDPN), lymphatic vessel hyaluronan receptor-1 (LYVE-1), VEGF-C, and VEGF-R3 [18]. Taken together, increased angio- and lymph-angiogenesis seems to be important for the pathophysiology and progression of CTCL, particularly MF.

The expression of CC chemokine receptor 7 (CCR7) is frequently up-regulated in advanced CTCL and is believed to be important in the spread of malignant T cells through dissemination to the sentinel lymph nodes and subsequently to the blood stream and internal organs [21, 22]. CC chemokine ligand 21 (CCL21), a ligand of CCR7, is up-regulated on lymphatic cells in the periphery by pro-inflammatory cytokines including membrane bound Lymphotoxin α (LTα) [23-25]. LTα is generally thought to be angio- and lymphangiogenic but, at higher concentrations it also display anti-vascular properties [26-29]. LTα promotes formation of lymph nodes, as shown in the LTα−/− mice [30], and plays an important role lymphangiogenesis [26]. LTα signals through TNFR1 and TNFR2, and the deregulation of TNFR signaling has previously been shown to protect malignant CTCL T cells from apoptosis [31-33]. Increased expression of LTα has been demonstrated in several types of cancer, including Burkitt's lymphoma [34, 35], and LTα polymorphisms are associated with increased risk of non-Hodgkin's lymphoma [36-39]. However, little is known about the role of LTα in the pathogenesis of CTCL.

Here we present evidence that malignant CTCL T cells strongly express LTα *in situ* and *in vitro* and that this expression is promoted by the aberrant activation of the JAK3/STAT5 pathway. We further demonstrate that through TNFR2, LTα functions as an autocrine factor by inducing production of IL-6. Finally, we show that LTα and IL-6 in concert with VEGF stimulate endothelial sprouting and tube formation. In summary, our observations indicate that LTα plays a role in angiogenesis and disease progression in CTCL.

## RESULTS

### LTα expression in CTCL

**Figure 1:**
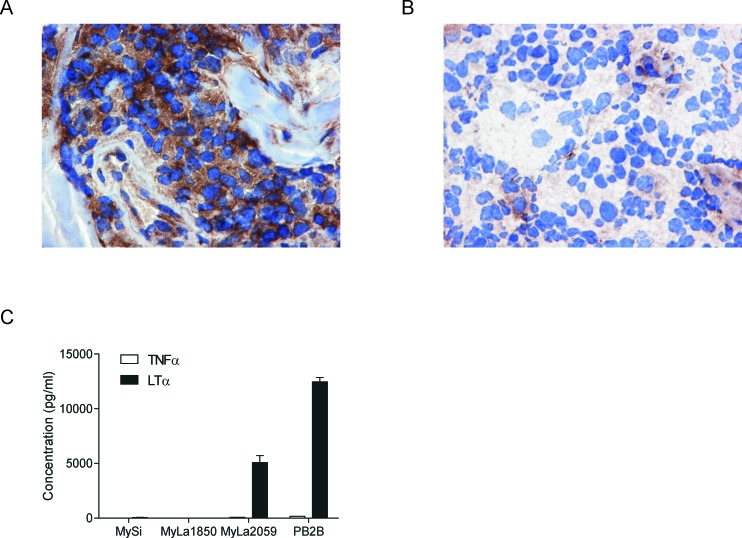
LTα expression in CTCL (**A**) Frozen biopsies of 10 patients diagnosed with CTCL (see Table 1) were subjected to IHC with an antibody directed against LTα showing strong cytoplasmic labelling of the neoplastic infiltrate in 8 of 10 cases (A × 400). (**B**) Shows an example of a patient staining negative for LTα (A × 400). (**C**) The expression of LTα and TNFα in non-malignant (MySi and MyLa1850) and malignant (MyLa2059 and PB2B) CTCL T cells lines were determined by ELISA. Bars represent mean values of three independent experiments.

To determine whether lymphoma T cells express LTα *in situ*, we performed immunohistochemical (IHC) staining of skin biopsy specimens obtained from 10 patients diagnosed with CTCL (Table 1). IHC staining with an anti-LTα antibody showed positive staining in eight out of ten patients (Figure 1A). Figure 1B shows an example of a patient staining negative for LTα. The staining was mainly cytoplasmic, although occasionally overlaying the nuclei. The intensity was moderate to strong in most patients with the most distinctive staining seen in medium-sized to large, neoplastic appearing cells. However, smaller lymphoid cells resembling reactive T cells also occasionally stained weakly positive (data not shown). In addition, IHC staining of skin biopsies from patients with benign inflammatory skin disorders stained negative for LTα (Supplementary Figure S1). In parallel, malignant T cell lines from CTCL patients displayed a constitutive synthesis of LTα as judged from the high concentrations (> 5 ng/ml) of the cytokine found in culture supernatants (Figure 1C). In contrast, non-malignant T cell lines established from skin and blood from CTCL patients did not spontaneously produce LTα as the cytokine was not detected in cell culture supernatants (Figure 1C) and the non-malignant T cells did not express LTα mRNA as measured by RT-PCR (data not shown). Neither malignant nor non-malignant CTCL T cells expressed significant levels of the closely related cytokine TNFα (Figure 1C).

**Table 1:** Patient characteristics and LTα and TNFR2 immunohistochemical stain status

Patient no.	Age at biopsy (years)	Sex	Diagnosis	LTα status in tumor cells	TNFR2 status in tumor cells
1	79	Male	MF	Positive	Positive
2	81	Female	MF	Positive	Positive
3	57	Male	MF	Positive	Positive
4	?	Male	MF	Positive	Positive
5	44	Male	SS	Positive	Positive
6	85	Male	NOS	Positive	Positive
7	54	Male	NOS	Negative	Positive
9	78	Male	NOS	Positive	Positive
8	63	Female	NOS	Negative	Positive
10	65	Male	ALCL	Positive	Positive

MF indicates Mycosis fungoides, SS: Sézary syndrome, NOS: not otherwise specified and ALCL: Anaplastic large cell lymphoma. MF patients were stage MF 1/2 (patch/plaque). Patients with SS, NOS or ALCL were all tumor stage.

### The oncogenic JAK3/STAT pathway induces expression of LTα

**Figure 2:**
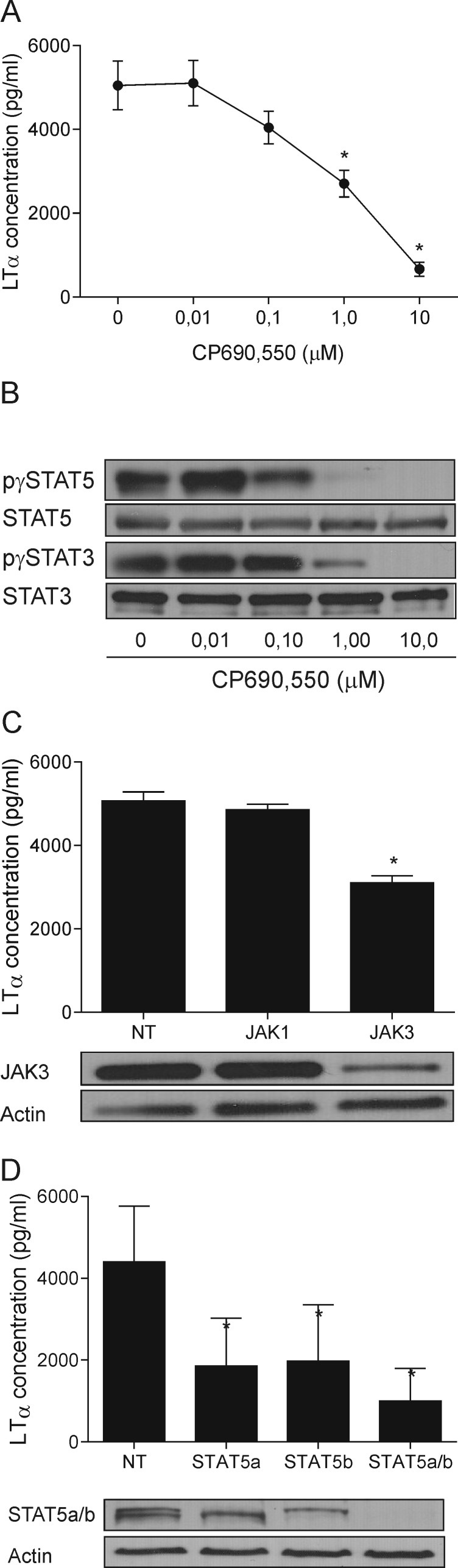
JAK3/STAT5 drives the LTα expression (**A**) LTα expression following inhibition of JAK activity in MyLa2059 with increasing concentrations of CP690,550 for 24 h *p < 0.05. (**B**) The phosphorylation status of STAT3 and 5 in MyLa2059 following incubation with CP690,550 for 24 h were analysed by WB. (**C, D**) MyLa2059 cells were transiently transfected with JAK1 and 3 (C) and STAT5a and b (D) or non-target (NT) control. 24h post-transfection, supernatants were harvested and LTα concentration measured by ELISA. Cells were lysed and the JAK3 (C) and STAT5 a and b (D) protein expression were analysed by WB. *p < 0.05 compared to NT control (paired *t*-test).

To address why malignant T cells spontaneously produce LTα, we focused on the JAK3/STAT3 pathway because this cell signal pathway promotes expression of several cytokines (IL-5, IL-10, IL-13, IL-17 and IL-17F) in CTCL cells [6-10]. As shown in Figure 2A and 2B, an inhibitor of JAK3 (CP690,550) suppress LTα production and STAT3 and STAT5 phosphorylation in a dose-dependent manner. Thus, a profound inhibition of LTα was observed at concentrations which also profoundly inhibited tyrosine phosphorylation of STAT3 (Figure 2B, lower part). Essentially, similar results were obtained in two other malignant T cell lines (PB2B and Mac2a) (Supplementary Figure S2). siRNA-mediated inhibition of JAK3 expression also resulted in a significant inhibition of LTα (Figure 2C) whereas siRNA against JAK1 had not effect indicating that JAK3 (and not JAK1) regulates LTα production in malignant T cells. As STAT5 is also a down-stream target of JAK3 and constitutively activated in malignant T cells [49], we examined the role of STAT5 in LTα production in malignant T cells. STAT5 tyrosine phosphorylation was inhibited in a dose-dependent manner by the JAK3 inhibitor (Figure 2B, upper part) and the inhibition correlated with inhibition of LTα (Figure 2, A *vs*. B upper part). Importantly, siRNA-mediated knock-down of STAT5a and STAT5b significantly inhibited LTα production by malignant T cells (Figure 2D). Of notice, a simultaneous knock-down of both STAT5a and STAT5b resulted in a complete inhibition of STAT5 expression and a profound inhibition of LTα (Figure 2D, right) indicating that both STAT5a and STAT5b are involved in the spontaneous LTα production. In contrast, siRNA mediated inhibition of STAT3 had no significant effect on LTα indicating that LTα expression is JAK3-dependent but STAT3-independent (Supplementary Figure S3).

### STAT5 binds to the promoter of the LTα gene

**Figure 3:**
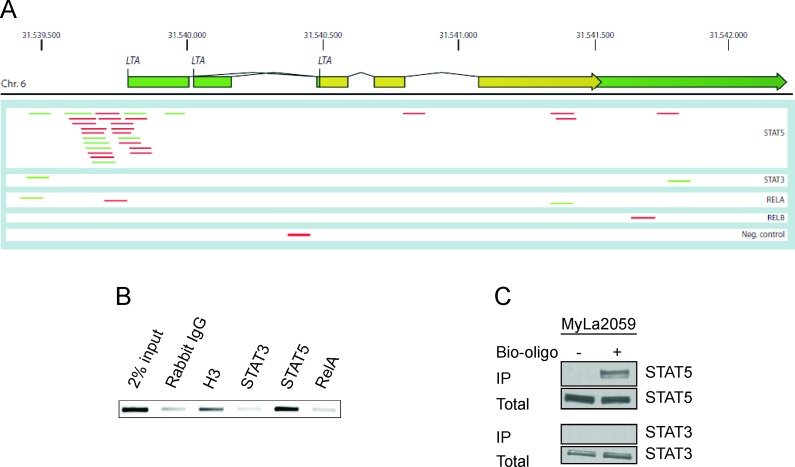
LTα is a transcriptional target of STAT5 (**A**) ChIP-seq reads from the *LTA* gene in malignant MyLa2059 cells. Reads (76 bases) obtained from immunoprecipitation of STAT5, STAT3, RELA, RELB and a negative control (rabbit IgG, bottom). The chromosomal position of *LTA* gene encoding LTα on chromosome 6 refers to *hg19*. Forward reads are indicated in green and reverse reads in red. (**B**) PCR analysis of ChIP samples using primers specific for the LTα promotor only detected the amplicon in the STAT5-precipitated sample, the 2% input, and the positive control (histone H3). (**C**) Pulldown assay using oligonucleotides representing a STAT binding site in the promoter region of the LTα gene. STAT5 binds to the sequence, while STAT3 do not.

Analyses of the LTα promoter region showed a number of binding sites for STAT and NFκB, which is also constitutively activated in malignant T cells [11, 14-17]. Accordingly, we performed chromatin immunoprecipitation followed by DNA sequencing (ChIP-seq) to identify whether LTα is a transcriptional target of STAT and Rel transcription factors in CTCL. ChIP-seq analysis of STAT5a/b-precipitated chromatin from malignant T cells yielded an enrichment of reads comprising a region of the LTα gene promoter (LTα, Figure 3A). In contrast, only a few reads for the LTα gene promoter were detected in chromatin precipitated with STAT3 antibody (Figure 3A). Likewise, precipitation with RelA and RelB did not yield significant number of reads for the LTα promoter (Figure 3A, lower, and data not shown). Next, we used the information from the ChIp-seq analysis to design primers flanking the STAT5-binding site in the promoter. These primers were subsequently used to detect enrichment by PCR of this specific sequence in chromatin precipitated from the malignant T cells with STAT5a/b and STAT3 antibodies relative to a negative control antibody (rabbit IgG). In keeping with the results above, PCR analysis of the precipitates showed enrichment of the sequence representing the LTα promoter in samples precipitated with the STAT5a/b antibody, whereas STAT3-precipitated samples were negative and equal to background levels (Figure 3B). Using an alternative, and less sensitive technique (Bio-oligonucleotide fishing), we also observed selective STAT5 binding to the LTα promoter. Thus, precipitation of STAT5 total cell lysates using bio-oligos corresponding to the sequence from −214 to −235 bp in the LTα promoter revealed a constitutive binding of STAT5 but not STAT3 in malignant T cells (MyLa2059) (Figure 3C). Essentially similar results were observed in another malignant CTCL cell line (Mac2a) (Supplementary Figure S4).

### TNF receptor expression in CTCL cells

**Figure 4:**
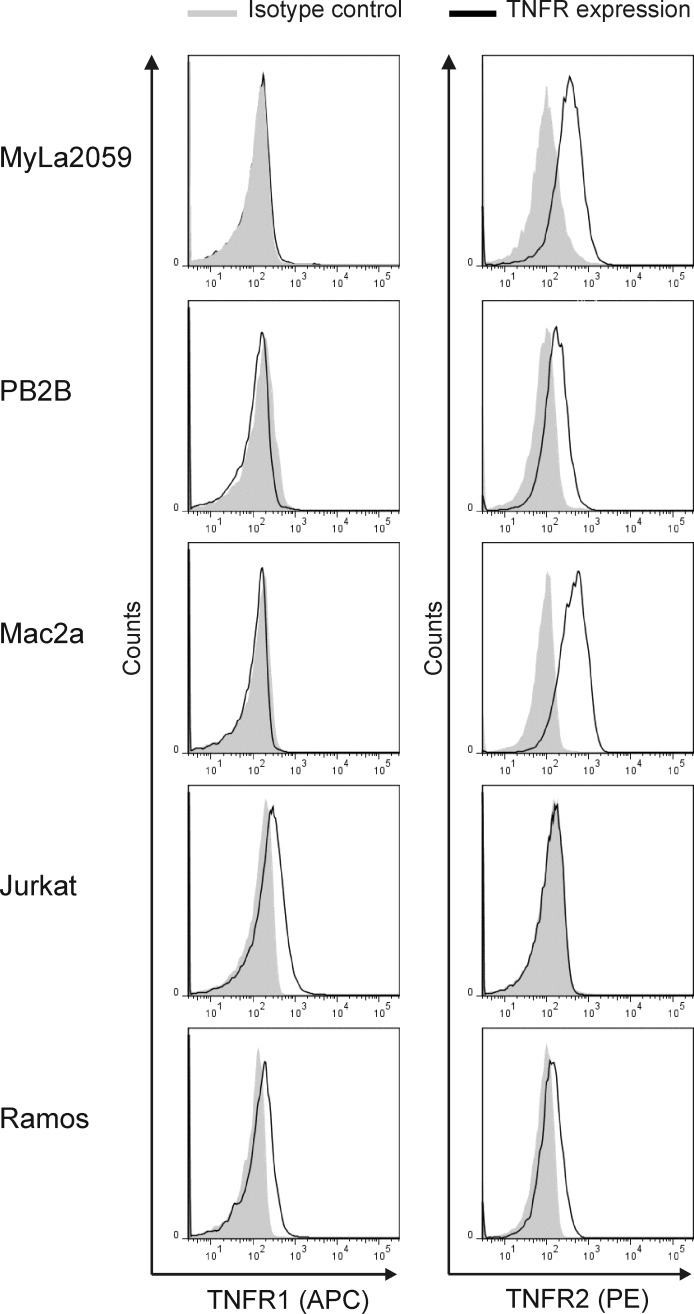
Expression of TNF receptors in CTCL Expression of TNFR1 and TNFR2 in malignant (MyLa2059, PB2B, Mac2a) CTCL T cells, Jurkat and Ramos were analysed by flowcytometry. Black line represents TNFR1 or TNFR2 expression, grey filled represent isotype control.

**Figure 5:**
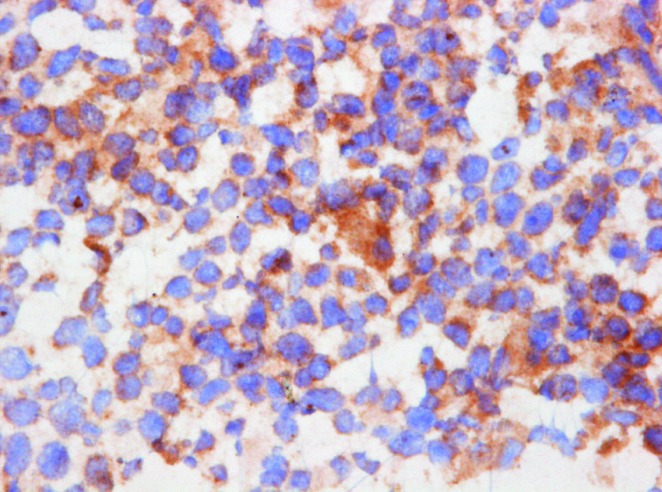
*In situ* TNFR2 expression in patients diagnosed with CTCL Immunohistochemical (IHC) staining of cryostat sections of CTCL with an antibody directed against TNFR2 showed cytoplasmic labelling of the neoplastic cells (A × 400) in all cases (see Table 1).

LTα signals through TNFR1 and TNFR2. The intracellular region of TNFR1 is characterized by the presence of a death domain close to its C-terminal end. Depending on the cellular context and the microenvironment conditions, TNFR1 activation can lead to transcription of pro-apoptotic and pro-necrotic genes [50]. On the other hand, the activation of TNFR2 mediates the transcriptional activation of genes related to cell proliferation and survival [50]. TNFR1 has previously been shown to be down-regulated in malignant CTCL T cells creating a possible way of protecting the malignant cells from TNF-induced apoptosis [31, 32]. To address whether LTα is an autocrine factor in CTCL, we examined the TNFR1 and TNFR2 expression in malignant CTCL T cells by flow cytometry. In line with the previous reports [31, 32], we found that malignant CTCL T cells do not express TNFR1, whereas the control, non-CTCL lymphoma cell lines Jurkat and Ramos were positive (Figure 4). Importantly, all of the malignant CTCL T cells tested (MyLa2059, PB2B and Mac2a) and the positive control (Ramos) expressed the alternative receptor, TNFR2 whereas the Jurkat cell line was negative as expected (Figure 4). To address whether TNFR2 expression was also seen *in situ*, we performed IHC staining with TNFR2 antibody of skin specimens from the ten patients examined in Figure 1. In agreement with the flow cytometry analyses of CTCL cell lines, specimens from all 10 patients (Table 1) stained positive for TNFR2 and expression was seen in both malignant T cells and stromal cells (Figure 5, and data not shown).

### LTα functions as an autocrine factor

**Figure 6:**
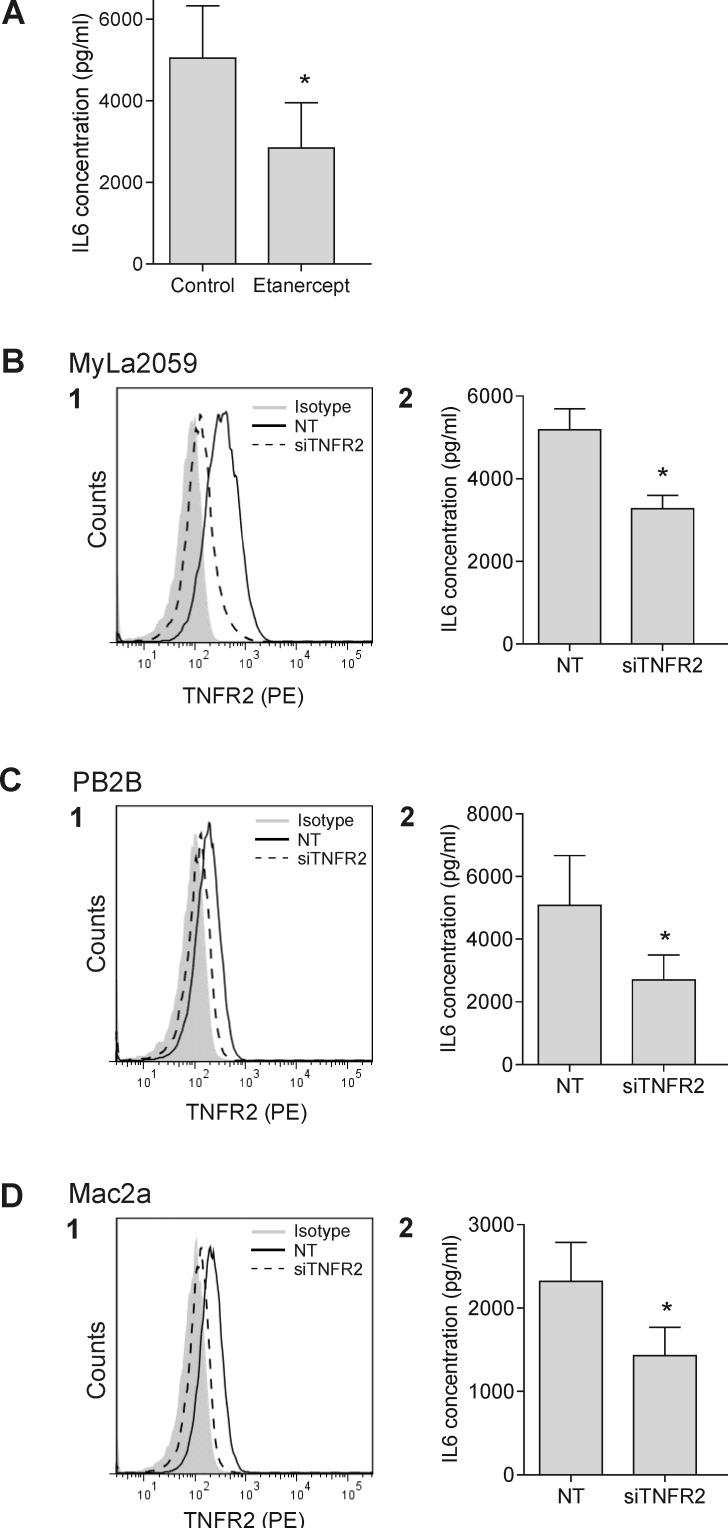
LTα regulates the IL-6 expression in malignant CTCL T cells (A) IL-6 expression in malignant CTCL cells (MyLa2059) were measured by ELISA following incubation with 100ug/ml Etanercept for 24 h. (B1, C1, D1) Selective down-regulation of TNFR2 with siRNA in malignant CTCL T cells (MyLa2059, PB2B and Mac2a). Filled grey represents isotype control, black line non-target (NT) control, and dashed line TNFR2 siRNA. (B2, C2, D2) IL-6 expression was measured after 24 h in MyLa2059, PB2B and Mac2a by ELISA following TNFR2 knock down. Bars represent mean values of three independent experiments. *p < 0.05 compared to NT (paired *t*-test).

To address whether LTα has an autocrine function in malignant T cells, we took two approaches. In the first, we took advantage of the human recombinant TNFR2/Fc fusion protein, Etanercept, to deplete LTα and in the second, we used siRNA to down-regulate TNFR2 expression. TNFR2 functionally linked to expression of inflammatory cytokines such as IL-1β and IL-6. As malignant T cells spontaneously synthesize IL-6 but not IL-1β (Figure 6 and data not shown), we examined the effect of Etanercept on the spontaneous production of IL-6 by malignant T cells. As shown in Figure 6A, LTα depletion by Etanercept diminished IL-6 concentration in supernatants by 45 % (Figure 6A, *p*<0.05, and Supplementary Figure S5). Targeting TNFR2 by siRNA significantly inhibited TNFR2 expression (Figure 6B-6D, left) and the spontaneous IL-6 production (Figure 6B-6D, right). Although only a partial knock-down of TNFR2 was observed, the spontaneous IL6 production was inhibited by 40-50% in all three malignant T cell lines (Figure 6B-6D) suggesting that LTα-TNFR2 signaling plays a key role in inducing IL-6 synthesis in malignant T cells. Surprisingly, Etanercept and siRNA TNFR2 knockdown did not affect the spontaneous proliferation of malignant T cells indicating that TNFR2 in these cells are only engaged in IL-6 production but not in mitogenesis and/or survival (Supplementary Figure S5, and data not shown). In addition, neutralization of LTα with Etanercept did not induce apoptosis in the malignant T-cells (Supplementary Figure S6).

### Malignant LTα and IL-6 promote endothelial cell sprouting

**Figure 7:**
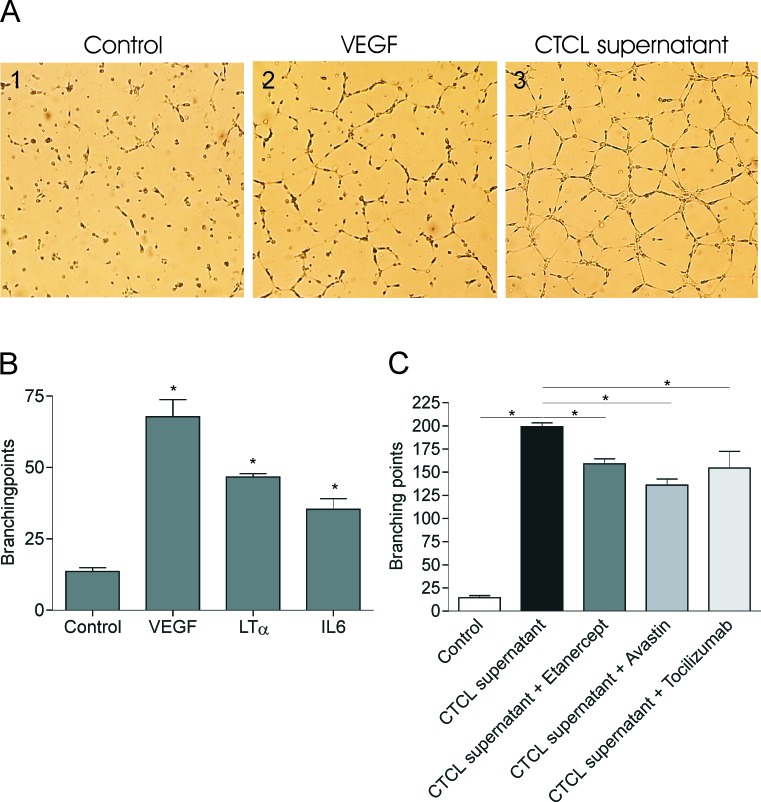
Malignant LTα and IL-6 promote endothelial cells sprouting Endothelial tube formation assays were performed on growth factor reduced matrigels in 24 well plates. (**A**) HUVEC cell sprouting when cultured in M200PRF alone (1), supplemented with VEGF (2) or malignant CTCL supernatant (1:2) (3). (**B**, **C**) Pictures were taken and number of branching points counted following treatment with human recombinant VEGF, LTα or IL-6 (**B**) or in the presence of malignant CTCL supernatant (1:2) either alone or supplemented with Avastin, Etanercept or Tocilizumab for 12 h. Bars represent mean values of three independent experiments. *p < 0.05 compared to control (paired t-test).

Recent observations indicate that lymphangio-/angio-genesis is involved in progression of CTCL [12, 18, 19, 51]. In addition to the well-established role in lymphoid organogenesis and lymphangiogenesis LTα can also induce angiogenesis. Likewise, IL-6 is a multifunctional cytokine that also has been demonstrated to play a role in angiogenesis and vascular remodelling [52-54]. In order to investigate the effect of malignant LTα and IL-6 on endothelial cell capillary morphogenesis we performed an endothelial cell tube formation assay using the matrigel platform. Figure 7A shows HUVEC cells cultured in medium (Figure 7A.1), in medium supplemented with VEGF-A as a positive control (Figure 7A.2), and in medium supplemented with culture supernatant isolated from malignant T cells (MyLa2059) (Figure 7A.3). HUVEC cells cultured en medium displayed small round shapes, isolated cells with only very few cells starting to align and no apparent tube formation (Figure 7A.1). As expected, cultures with VEGF-A displayed cell migration, alignment, and rudimentary, partial capillary structures without complete tube formation (Figure 7A.2). Importantly, HUVEC cells exposed to culture supernatant from malignant T cells displayed extensive endothelial cell migration, elongation, alignment and branching with tube formation in the form of closed polygons and complex mesh-like structures (Figure 7A.3) indicating that malignant T cells produce factors which are potent inducers of angiogenesis. Figure 7B shows the response of HUVEC cells to human recombinant VEGF-A, LTα, and IL-6 confirming that HUVEC cells respond to each of these factors produced by the malignant T cells. To address contribution of the various factors in the tumor cell supernatants to the endothelial cell response, HUVEC cells were incubated with CTCL cell culture supernatants supplemented with or without inhibitors of each cytokine. HUVEC cells plated with malignant supernatant in the presence of anti-VEGF (Avastin), anti-LTα (Etanercept), or anti-IL-6 receptor (Tocilizumab) was clearly affected by the neutralizing antibodies/fusionprotein. Thus, the characteristic mesh-like structures and number of branching points induced by CTCL supernatant (Figure 7A and Figure 7C, black bar) were significantly decreased when the cultures were spiked with Avastin, Etanercept, and Tocilizumab, respectively (Figure 7C). These observations indicate that malignant CTCL T cells stimulate endothelial sprouting and tube formation through the secretion of LTα and IL-6 in concert with VEGF.

## DISCUSSION

LTα has been implicated in several diseases including various malignancies. Yet, little is known about its expression and function in CTCL. In the present study, we provide evidence that LTα and TNFR2 are expressed in malignant T cell lines and lymphocytes with malignant cell morphology *in situ* in skin lesions suggesting that LTα plays a role in the pathogenesis of CTCL. Importantly, the production of LTα was induced by STAT5, which promoted proliferation and drug resistance in CTCL [55]. In normal T cells, LTα is seemingly induced by IL-2 through a JAK3-mediated activation of STAT5a [56], whereas our findings suggest that both STAT5a and STAT5b are involved in malignant LTα production. Thus, STAT5 binds the LTα promoter and siRNA-mediated knockdown of STAT5a and STAT5b inhibited the spontaneous LTα production by malignant T cells. In accordance, both forms of STAT5 proteins are constitutively activated by JAK3, which is constitutively active in malignant T cells due to a loss of regulatory control by the protein tyrosine phosphatases SHP1 and SOCS3 [57]. Interestingly, STAT3 is also activated by JAK3 in these T cells [58], but not involved in the regulation of the malignant LTα production. The NFκB signaling pathway is also aberrantly activated in malignant T cells but despite the well-established role of NFκB as an inducer of LTα in other cells [59], NFκB proteins did not bind the LTα promoter indicating that this pathway was not involved in malignant LTα production. Thus, these findings indicate that the regulation of LTα expression was highly specific and that the JAK3/STAT5 pathway plays a unique role in LTα transcription in malignant T cells. Taken together, the present findings add significantly to the accumulating body of evidence that the aberrant activation of the JAK3/STAT signaling pathway induce expression of multiple cytokines by malignant T cells. Accordingly, the JAK3/STAT pathway induce synthesis of IL-5, IL10, IL-13, IL-17A, IL-17F and now LTα, all of which have been directly or indirectly implicated in the pathogenesis of CTCL [6-10]. The present findings strengthen the hypothesis that JAK3 is an attractive target for future therapy of CTCL patients with small molecule JAK inhibitors.

As malignant T cells expressed TNFR2, we hypothesized that LTα functions as an autocrine activator of malignant T cells. Under certain inflammatory conditions, LTα triggers the production of IL-6 and activation of TNFR2 has also been shown to initiate the IL-6 production [60, 61]. Since malignant T cells spontaneously produce significant amounts of LTα and IL-6 [10], we questioned whether LTα was implicated in aberrant synthesis of IL-6 in CTCL cells. To this end, we used two complementary approaches: first, we took advantage of an LTα-binding fusion protein, Etanercept, which binds LTα with high affinity, to neutralize and remove soluble LTα. Second, we used siRNA-mediated depletion of TNFR2 in the malignant T cells. Importantly, both approaches yielded almost identical results, namely a significant inhibition of the spontaneous IL-6 production by malignant T cells. The IL-6 synthesis was only partly inhibited (40-50%) suggesting that other factors in addition to LTα were also involved. Alternatively, limitations in our assays and procedures – such as an incomplete siRNA-mediated gene knockdown – might also explain why we only observed a partial suppression of the IL-6 production. Despite such limitations, our data clearly and consistently shows that LTα is an autocrine inducer of the spontaneous IL-6 production by CTCL cells.

Similar to other malignancies, the formation of blood and lymphatic vasculature has been recently hypothesized to play a key role in the pathogenesis of CTCL [12, 18, 19]. In support of this notion, an increase in angiogenesis and lymphangiogenesis correlates with CTCL progression [18, 19]. Moreover, malignant T cells produce angiogenic factors such as VEGF-A and VEGF-C *in vitro* and *in vivo* [12, 51]. As LTα and IL-6 are both capable of directly and indirectly induce angiogenesis and lymphangiogenesis, we hypothesized that CTCL-derived LTα and IL-6, in addition to VEGF [12] would stimulate pathological neovascularization in the CTCL lesions. Accordingly, we examined the effect of culture supernatants from malignant T cells on HUVEC cells in an *in vitro* model of angiogenesis and, indeed, these supernatants stimulated migration, elongation, branching, and tube formation of the HUVEC cells. Importantly, the angiogenic response was partly inhibited by the addition of anti-VEGF (Avastin), anti-LTα (Etanercept), or anti-IL-6 receptor (Tocilizumab) agents, suggesting that malignant T cells – at least *in vitro* – drive angiogenesis through secretion of VEGF, LTα, and IL-6. Yet, the present results indicate the LTα directly - and indirectly via induction of IL-6 - contributes to the angiogenic response elicited by the CTCL cell culture supernatants. Since lymphocytes with malignant cell morphology express VEGF-A [12], VEGF-C [51], IL-6 [10] and LTα (as shown here) *in situ* in CTCL skin lesions, and all of these factors are well established drivers of tumor associated (lymph)angiogenesis, our data suggest that malignant T cells orchestrate neovasculization in CTCL and, thus, contribute to the disease progression.

In conclusion, we provide the evidence that the JAK3/STAT5 pathway drives an aberrant expression of LTα in CTCL. We further show that LTα acts as an autocrine factor stimulating IL-6 expression and, together with IL-6 and VEGF-A, it stimulates endothelial sprouting and tube formation. These data strongly suggest that LTα is involved in the pathogenesis of CTCL and may serve as a potential direct or indirect therapeutic target.

## MATERIALS AND METHODS

### Antibodies and reagents

The antibody against total STAT5a/b was from BD Transduction Laboratories (Franklin Lakes, NJ, USA), while antibodies against pY-STAT5, total STAT3 and pY-STAT3 were all from Cell Signaling Technology (Beverly, MA, USA). JAK3 was from Santa Cruz Biotechnology (Santa Cruz, CA, USA), and α-actin was from Sigma-Aldrich (St Louis, MO, USA). The Jak3 inhibitor CP-690550 and Etanercept were from Pfizer (Ballerup, Denmark), Tocilizumab and Avastin were from Roche (UK). DMSO was from Sigma-Aldrich (St Louis, MO, USA) and recombinant human (rh) LTα, rhTNFα and rhIL-6 were from RnD systems (R&D Systems Europe Ltd, Abingdon, UK).

### Cell lines and cell culture

The malignant T cell line MyLa2059 and PB2B and the non-malignant T cell lines MyLa1850 and MySi were generated from patients with MF [40-42]. Mac2a is a malignant anaplastic large-cell lymphoma (ALCL) cell line established from a skin tumor in the progressive phase of the disease [43] The Jurkat T cell line, J-Tag [44], and the B-cell line, Ramos 2G6 [45], have been described elsewhere. MyLa2059, PB2B, Mac-2a and Ramos were grown in conditional media (CM) (RPMI 1640, 2 mm l-glutamine and 100 g/ml penicillin/streptomycin; all from Sigma) supplemented with 10% fetal bovine serum (Life Technologies, Denmark). The MyLa1850 and MySi cell lines were grown in CM supplemented with 10% pooled human serum (HS) (Blood Bank, State University Hospital, Copenhagen, Denmark) and 10^3^ U/ml interleukin (IL)-2 (Proleukin™). Primary human umbilical vein endothelial cells (HUVEC), pooled from multiple donors, were purchased from Life Technologies, Denmark. All experiments were performed with cells at passage 2 to 4. HUVEC cells were cultured in Medium 200PRF (Life Technologies, Denmark) supplemented with low serum growth supplement (LSGS) (Life Technologies, Denmark).

### Patients

The study includes frozen biopsies from 10 patients diagnosed with CTCL during the period 1979-2004. Samples were drawn from the frozen tissue bank at the Department of Pathology at Rigshospitalet [46]. For immunohistochemistry, 4 cases with MF, 1 case of SS, 1 case of primary cutaneous anaplastic large cell lymphoma (ALCL), and 4 cases of primary cutaneous peripheral T-cell lymphoma, not otherwise specified (PTL, NOS) were selected for analysis. The patients were 8 males and 2 females with a median age of 65 years (range 44-85 years) at diagnosis. Controls consisted of lymphoid organs with benign hyperplasia, and 9 frozen biopsies from patients with benign skin inflammatory disorders.

### Immunohistochemistry

Cryostat sections were air-dried, fixed in acetone for 10 min and incubated overnight at 4 degree C with anti LTα (Sigma Prestige) or TNFR2 (RnD Systems) both diluted 1:20. The sections were washed and developed using HRP-conjugated secondary antibody and DAB. Sections were counterstained with haematoxylin, mounted and examined in an Olympus BX51 microscope equipped with Colour View soft imaging system for photomicrographs. Positive controls were performed by staining lymph node with benign hyperplasia and negative controls by omitting the primary antibody (data not shown).

### Enzyme-linked immunosorbent assay

Cells (1×10^6^ cells/sample) were washed and resuspended in 3 ml fresh cytokine-free media in a 6-well plate. Subsequently, the cells were incubated for 24 hours with or without the given concentrations of inhibitors or siRNA. Finally, the supernatants were harvested and the concentrations of TNFα, LTα or IL-6 were measured by enzyme-linked immunosorbent assay (ELISA) using a human TNFα-, LTα- or IL-6 specific ELISA kit (R&D Systems Europe Ltd, Abingdon, UK). These assays employ the quantitative sandwich ELISA technique. Optical densities were measured at 450 nm using a multiscan FC microplate reader (ThermoScientific).

### Protein extraction and western blotting

1-2×10^6^ cells were rapidly pelleted and lysed in ice-cold lysis buffer and subjected to sodium dodecyl sulfate-polyacrylamide gel electrophoresis and western blotting as previously described [47].

### Chromatin immuneprecipitation (ChIP)

SimpleChip^®^ Enzymatic Chromatin IP kit (Agarose Beads) from Cell Signalig Technologies was used according the manufacturer's protocol and is described in detail elsewhere^11^. All antibodies (Rabbit IgG, Histone H3, STAT3, STAT5, RelA, and RelB) were from Cell Signaling Technologies. Immunoprecipitated and input DNA were subjected to PCR using PCR primers for LTα promoter upstream regions flanking STAT5 binding sites: LTα promoter forward: 5′-TGATTGCTCTTCAGGGAACC, and reverse: 5′-TGAGGCCTAGGAGAGAACCA as described previously [9]. PCR annealing temperature was 58°C, and the amplicon size was 202 bp. Library construction for ChIP sequencing was performed by usage of the Illumina's “ChIP-Seq DNA Sample Prep Kit, IP-102-1001” with 10 ng of immunoprecipitated DNA as starting material and with gel size selection of the 150-250 bp after adaptor ligation. Sequencing was performed as single read sequencing (76 cycles) on an Illumina GAIIx. ChIP-seq reads were aligned to *hg19* (on average 21 million reads/library) using CLC Genomics Workbench.

### Oligonucleotide affinity purification

To study DNA binding protein, an oligonucleotide affinity purification experiment was performed based on a biotin-avidin affinity system. 10×10^6^ cells/sample were lysed in ice-cold lysis buffer and pre-cleared for 3 hours with avidin-agarose beads. 125 pmol biotinylated oligonucleotides (bio-oligos) with the sequence F: 5′-CTCCCTTTCCCAGAACTCAGT and R: 5′-ACTGAGTTCTGGGAAAGGGAG was added together with avidin-agarose beads and incubated overnight at 4°C while rotating. Then, the beads were thoroughly washed with lysis buffer and subsequently prepared for SDS-PAGE followed by Western-blotting as previously described [47].

### Transient transfections

Small interfering RNA (siRNA) transfection was performed by using Amaxa Nucleofector technology (Amaxa GmbH, Cologne, Germany), using 0.5 nmol of siRNA against JAK3, STAT5a or STAT5b and non-targeting (NT) control #1 (ONtarget PLUS, smart pool, Dharmacon, Chicargo, IL, USA) or siRNA directed against TNFR2 and universal negative control #1 (sigma) on 2×10^6^ cells, described in detail previously [48].

### Flow cytometry

For surface staining of TNFRs, cells were harvested, washed in ice-cold FACS buffer and stained with 1 μl APC-conjugated anti-TNFR1, PE-conjugated anti-TNFR2 or isotype control Abs (all from R&D Systems) for 30 min at 4°C in the dark. After final washing, the cells were resuspended in FACS buffer and analyzed on a FACSCalibur or a LSRII flow cytometer (BD Biosciences). FlowJo software (Tree Star, Ashland, OR, USA) was used for data analysis.

### Matrigel endothelial tube formation assay

To study the effect of LTα produced by CTCL T cells in an *in vitro* model of angiogenesis, a matrigel endothelial tube formation assay was performed. The endothelial cell tube formation assay was performed on growth factor-reduced matrigel (Geltrex™ Matrix, Life Technologies) in which the levels of stimulatory cytokines and growth factors have been markedly reduced. Pre-cooled 24 well plates were coated with 100 μl Geltrex™ and left for 30 min to allow the gel to solidify. HUVEC (2,5×10^4^ cells per well) were plated on the matrigel-coated culture plates in M200RPF endothelial culture medium alone (control), with the addition of 10 pg/ml rhVEGF-A (positive control), or 10 pg/ml recombinant human LTα, TNFα or IL-6. In other experiments, HUVEC cells were cultured in the presence of malignant CTCL culture supernatants (1×10^6^ MyLa2059/3ml/24h) (1:2) for 10 hours, either alone or supplemented with 100 μg/ml Etanercept, 25 μg/ml Avastin or 20 μg/ml Tocilizumab for 10 hours. Photographs (x4) were taken, and the number of branching points was counted.

### Statistics

Data are reported as mean plus or minus SEM, and the differences were evaluated by Student's *t*-test. All experiments were repeated at least 3 times and all data were pooled for statistical analysis. P values <0.05 were considered to be statistically significant. Statistical analysis was performed in GraphPad Prism Version 4.03 software (GraphPad Software Inc., San Diego, CA or Excel (Microsoft Corp., Redmond, WA).

## SUPPLEMENTARY MATERIAL FIGURES


