# Supplementary material for: Malignant T cells express lymphotoxin α and drive endothelial activation in cutaneous T cell lymphoma
Source: Oncotarget. 2015 Apr 15;6(17):15235–49. doi: 10.18632/oncotarget.3837 (PMC4558148; doi:10.18632/oncotarget.3837)
Supplement: Supplementary file 1 [file oncotarget-06-15235-s001.pdf]

Malignant T cells express lymphotoxin α and drive endothelial activation in cutaneous T cell lymphoma

Supplementary Material

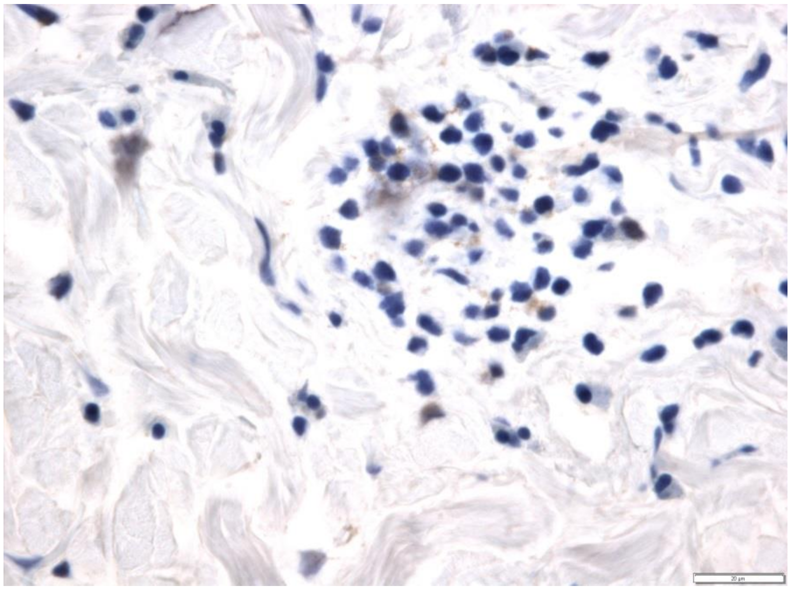

Figure S1: LTα in not expressed in benign inflammatory skin disorders. Representative frozen biopsy from a patient with benign skin inflammation was subjected to IHC with an antibody directed against LTα showing negative staining of perivascular small lymphocytes. Essentially similar results were found in 9 out of 9 cases.

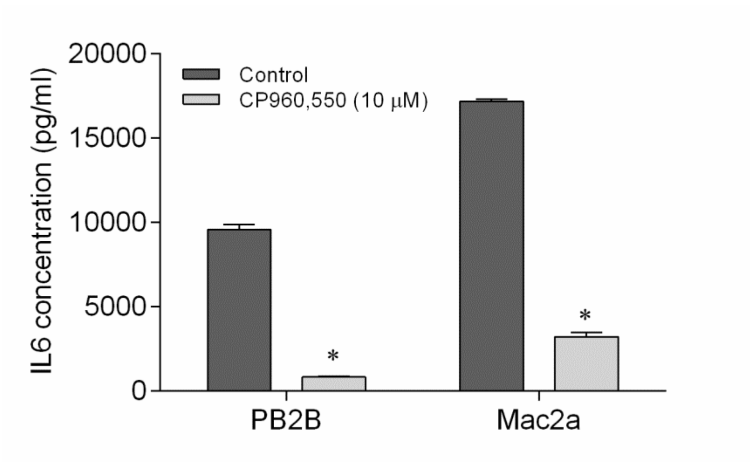

Figure S2: JAK regulate LTα expression in malignant CTCL T cells. Two malignant CTCL T cell lines (Mac-2a and PB2B) were incubated with 10μM CP6990,550 for 24h. Subsequent the concentrations of LTα in the culture supernatants were measured by an LTα specific ELISA. Bars represent mean values of three independent experiments. *p<0.05 compared to control (paired t-test).

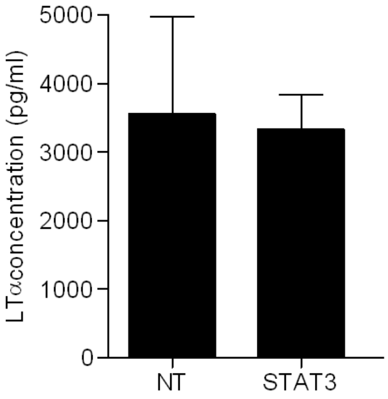

Figure S3: STAT3 do not regulate the LTα expression in CTCL. MyLa2059 cells were transiently transfected with STAT3 siRNA. 24 hours post-transfection, supernatants were harvested and LTα concentration measured by ELISA. Bars represent mean values of three independent experiments.

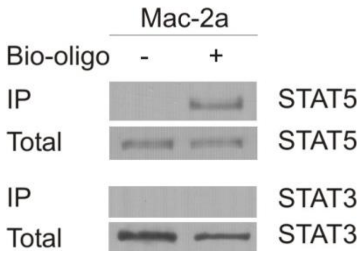

Figure S4: STAT5 bind to the LTα promoter region. Pull-down assay using oligonucleotides representing a STAT binding site in the promoter region of the LTα gene. STAT5 bind to the sequence, while STAT3 do not.

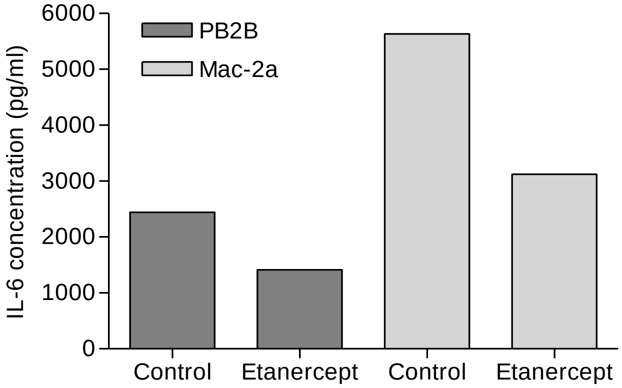

Figure S5: LTα regulates the IL-6 expression in malignant CTCL T cells. The IL-6 expression in two malignant CTCL cell lines (Mac-2a and PB2B) was measured by ELISA following incubation with 100ug/ml Etanercept for 24 h. Bars represent one experiment.
